# The characteristics and patterns of e-cigarette use and its association with cigarette cessation intention in a Chinese smoking population: A mediation analysis

**DOI:** 10.18332/tid/144251

**Published:** 2022-02-11

**Authors:** Hao-Xiang Lin, Yan Zhang, Mei-Jun Chen, Yun-Ting Zheng, Qing-Ping Yun, Lan-Chao Zhang, Wan-Tong Zhang, Bao-Chen Zhu, Zhao Liu

**Affiliations:** 1 Department of Social Medicine and Health Education, School of Public Health, Peking University Health Science Center, Beijing, China; 2 China Journal of Traditional Chinese Medicine and Pharmacy, Beijing, China; 3 Institute of Clinical Pharmacology, Xiyuan Hospital, China Academy of Chinese Medical Sciences, Beijing, China; 4 Department of Pharmacology, Dongzhimen Hospital, Beijing University of Chinese Medicine, Beijing, China; 5 Tobacco Medicine and Tobacco Cessation Center, China-Japan Friendship Hospital, Beijing, China

**Keywords:** electronic cigarettes, mediation analysis, quitting intention

## Abstract

**INTRODUCTION:**

The use of e-cigarettes has become more common in China, but the research on e-cigarettes in China, while growing, is still limited. This study examined the characteristics and patterns of e-cigarette use, and analyzed the possible mediators between cigarette cessation intention and e-cigarette use in a Chinese smoking population.

**METHODS:**

This was a cross-sectional study conducted in mainland China. By convenience sampling method, the participants were recruited from 85 major commercial streets of several large cities in China. The study interviewers completed face-to-face interviews and uploaded the completed questionnaires into the online survey platform. The participants were contacted for clarification if any problems were detected. Logistic regression yielded adjusted odds ratios (ORs) for ever use of e-cigarettes. We further conducted a mediation analysis to estimate the effect of possible mediators.

**RESULTS:**

From July to August 2020, a total of 738 smokers were invited to participate in this study; 613 smokers were identified as eligible and 609 smokers were included in this analysis. Of them, 24 (3.94%) participants were currently using e-cigarettes, and 165 (27.09%) participants have ever used e-cigarettes. The participants with younger age were more likely to have ever used e-cigarettes, ranging from 37.5% in the 18–29 years age group to 6.5% in the 60–69 years age group. After controlling for demographic characteristics and nicotine dependence, the ever use of e-cigarettes was significantly associated with younger age, higher education level, higher monthly income, previous smoking cessation attempts and quitting intention. With the mediation analysis, the education level is confirmed as a mediating factor, and approximately 42.86% of the effects were mediated through the channel of higher socioeconomic status.

**CONCLUSIONS:**

This is the first study to examine the possible mediators between cigarette cessation intention and e-cigarette use in a Chinese smoking population. The findings revealed that high socioeconomic status, particularly higher education level, was a major mediating factor.

## INTRODUCTION

Electronic cigarettes (e-cigarettes), more accurately known as ‘electronic nicotine delivery systems (ENDS)’, have emerged as a new type of tobacco product^1^. Since marketed nearly two decades ago, the use of e-cigarettes has grown rapidly, particularly in Europe and USA^2,3^.

China is the largest tobacco-consuming country in the world, and approximately 80% of the world’s e-cigarettes are produced in China^4^. Although the use of e-cigarettes in China is currently lower than that in developed countries, it has become increasingly popular. The China Adult Tobacco Survey in 2015 and 2018^5,6^ showed that 48.5% of the population aged ≥15 years heard of e-cigarettes in 2018, up from 40.5% in 2015; 5.0% tried e-cigarettes at least once in their lifetime in 2018, up from 3.1% in 2015. Moreover, current use of e-cigarettes had almost doubled, from 0.5% to 0.9%.

The reasons for the rapid increase of e-cigarette use in China include the large number of smokers, the growing concerns about harms of cigarette smoking, the implementation of strong smoke-free legislations, and the aggressive marketing activities of e-cigarette manufacturers^7^. More importantly, the most common reason for using e-cigarettes in China is smoking cessation^8-10^, although there are different opinions on the level of evidence for smoking cessation^11^.

As the use of e-cigarettes becomes more common in China, there is a need for a more detailed assessment of the characteristics, patterns and associated factors of e-cigarette use. Unfortunately, the research on e-cigarettes in China, while growing, is still limited. Therefore, to help fill the evidence gap, this study aimed to examine the characteristics and patterns of e-cigarette use, and to analyze the possible mediators between cigarette cessation intention and e-cigarette use in a Chinese smoking population.

## METHODS

### Study design and participants

This was a cross-sectional study conducted in July and August 2020 in China, details of which have been reported elsewhere^12^. In brief, this study was divided into four stages. In the first stage, experts at China-Japan Friendship Hospital and Peking University designed the survey questionnaires, and an online survey platform was developed based on several recommendations from our previous studies^13^. For example, this online survey platform was programmed in PL/SQL on an Oracle 8 database server for more efficient management; HTML5 was chosen as the basis for better displays; the total length of the program was 2363 lines and the file sizes were 105 kb to minimize the download time. The whole programming, testing and verification took approximately 40 hours.

In the second stage, the research group provided online training for all study interviewers, including methods of obtaining informed consent from all participants, valuable skills when conducting interviews (such as how to question effectively and how to make participants comfortable), and other skills (such as how to approach more participants, keep study interviewers safe from COVID-19 and deal with problems if participants were unhappy).

In the third stage, all the study interviewers completed face-to-face interviews. Participants were randomly selected on public streets. A total of 85 major commercial streets of several China large cities (such as Beijing, Hangzhou, Xi’an, Wuhan, Chongqing, etc.) were selected. There were no special criteria of selecting the streets. No borders are involved. No street was revisited. Each interview lasted for approximately 15–20 min. The participants were recruited based on the following criteria: 1) aged 18–70 years; 2) current smokers; 3) local residents; and 4) consented to participate in the study. The questionnaires were completed by the respondents themselves. If the respondents could not complete the questionnaire on their own, the study investigators helped them to complete the questionnaire.

In the last stage, the interviewers recorded the completed questionnaires into the online survey platform; at the same time, all submitted questionnaires were reviewed by the research group and participants were contacted for clarification if any problem was detected.

### Measures

The demographic questionnaire collected sociodemographic information, including age, gender, educational level and monthly income. The questionnaire on smoking was adapted from the Global Adult Tobacco Survey (GATS)^14^ and China Adult Tobacco Survey^5,6^. For this study, current cigarette/e-cigarette smoking was defined as currently smoking cigarettes/e-cigarettes at the time of survey. All the current cigarette smokers were asked what types of cigarettes they smoked, the age they started cigarette smoking, the duration of cigarette smoking, and the number of cigarettes smoked per day. Ever use of e-cigarettes was defined as self-reported ever use of e-cigarettes for at least once in their lifetime, regardless of types, amount and duration of e-cigarettes.

According to China Clinical Smoking Cessation Guideline^15^, nicotine dependence was identified if a minimum of 3 of the following 6 were met: 1) craving or a persistent desire or urge to use tobacco; 2) a persistent desire or unsuccessful efforts to cut down or control tobacco use; 3) tobacco withdrawal (such as irritability, frustration, anger, anxiety, difficulty concentrating, increased appetite, restlessness, insomnia) after abrupt cessation of tobacco use, or reduction in the amount of tobacco used; 4) tolerance, defined as the need for markedly increased amounts of tobacco to achieve the desired effect; 5) important social, occupational, or recreational activities are given up or reduced because of tobacco use; and 6) tobacco use is continued despite knowledge of having a persistent or recurrent physical or psychological problem that is likely to have been caused or exacerbated by tobacco.

### Statistical analysis

Descriptive analyses were used to describe the characteristics of the study population. We assessed the significance of differences by ANOVA or Student’s t-test for continuous variables and χ^2^ test for categorical variables. The logistic regression analysis was applied to identify the associated factors of ever use of e-cigarettes, and odds ratios (OR) and 95% confidence intervals (CIs) were obtained. Model 1 adjusted for gender and age, while Model 2 adjusted for gender, age, ethnicity, education level, monthly income, chronic disease, self-reported overall satisfaction of life, alcohol use, nicotine dependence, previous smoking cessation attempts, and secondhand smoke exposure.

The mediation analysis was conducted in three steps^16^. First, we employed binary logistic regression to examine the direct effects of ever use of e-cigarettes to confirm its effectiveness (direct effect). Second, we used a structured equation modeling (SEM) approach to test the indirect effects. We then performed a Sobel test to estimate the mediation effect of education and income.

The SPSS 19.0 was used for logistic regression, AMOS 24.0 for SEM, and STATA 14.0 for mediation analysis. All reported p-values were two-sided, and significance was set at p<0.05.

## RESULTS

**Figure 1:**
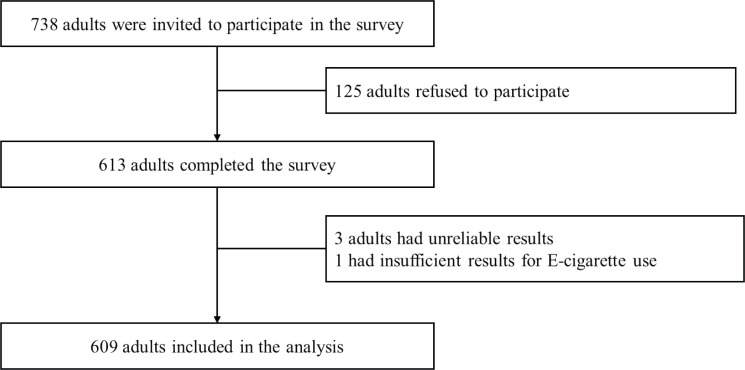
Flow chart of selection of participants

In July and August 2020, 738 smokers were invited to participate in the study. After screening, 613 smokers were identified as eligible for further interviews. However, 4 participants had unreliable results related to e-cigarette use or smoking status, and were excluded from the analysis (Figure 1). As such, 609 current smokers were included into this analysis. The descriptive statistics for the overall study population is shown in Table 1. Of the 609 smokers analyzed, 91.6% were men, mean age (SD) was 37.95 (14.31) years, and 58.5% had an educational level of college or higher; the mean (SD) cigarettes smoked per day was 10.62 (9.73), the mean (SD) smoking duration was 17.32 (13.01) years, and 39.2% of the study population had nicotine dependence. In this study population, 24 (3.94%) participants were currently using e-cigarettes, and 169 (27.09%) participants had ever used e-cigarettes.

**Table 1:** Demographics of study population

*Variables*	*Never use of e-cigarettes n (%)*	*Ever use of e-cigarettes n (%)*	*p*	*Total n (%)*
**Total**, n	440	169		609
**Gender**			0.447	
Male	404 (91.8)	154 (91.1)		558 (91.6)
Female	36 (8.2)	15 (8.9)		51 (8.4)
**Age** (years)			**0.000**	
18–29	148 (33.6)	90 (53.3)		238 (39.1)
30–39	61 (13.9)	25 (14.8)		86 (14.1)
40–49	101 (23.0)	32 (18.9)		133 (21.8)
50–59	102 (23.2)	20 (11.8)		122 (20.0)
60–69	28 (6.4)	2 (1.2)		30 (4.9)
Mean (SD)	39.82 (14.75)	33.04 (11.77)		37.95 (14.31)
**Ethnicity**			0.169	
Han	394 (89.5)	146 (86.4)		540 (88.7)
Other	46 (10.5)	23 (13.6)		69 (11.3)
**Education level**			**0.000**	
Primary school or less	42 (9.5)	4 (1.8)		45 (7.4)
Middle and high school	165 (37.5)	49 (25.4)		208 (34.2)
College or higher	233 (53.0)	116 (72.8)		356 (58.5)
**Personal monthly income** (RMB)			**0.011**	
<3000	126 (28.6)	47 (27.8)		173 (28.4)
3000–5999	177 (40.2)	50 (29.6)		227 (37.3)
6000–9999	86 (19.5)	34 (20.1)		120 (19.7)
>10000	51 (11.6)	38 (22.5)		89 (14.6)
**Chronic disease**			0.230	
Yes	78 (17.7)	25 (14.8)		103 (16.9)
No	362 (82.3)	144 (85.2)		506 (83.1)
**Overall satisfaction of life**			0.101	
Happy	21 (4.8)	7 (4.1)		28 (4.6)
Moderate	144 (32.7)	43 (25.4)		187 (30.7)
Not Happy	275 (62.5)	119 (70.4)		394 (64.7)
**Alcohol use**			0.450	
Yes	337 (76.6)	131 (77.5)		468 (76.8)
No	103 (23.4)	38 (22.5)		141 (23.2)
**Cigarettes smoked per day**			0.343	
1–9	217 (49.3)	85 (50.3)		302 (49.6)
10–19	102 (23.2)	48 (28.4)		150 (24.6)
≥20	121 (27.5)	36 (21.3)		157 (25.8)
Mean (SD)	10.73 (9.65)	10.33 (9.98)		10.62 (9.73)
**Smoking duration** (years)			0.091	
1–9	161 (36.6)	49 (29.0)		210 (34.5)
10–19	89 (20.2)	37 (21.9)		126 (20.7)
≥20	190 (43.2)	83 (49.1)		273 (44.8)
Mean (SD)	16.71 (12.71)	18.91 (13.67)		17.32 (13.01)
**Nicotine dependence**			**0.022**	
Yes	184 (41.8)	55 (32.5)		239 (39.2)
No	256 (58.2)	114 (67.8)		370 (60.8)
**Secondhand smoke exposure**				
Yes	174 (39.5)	63 (37.3)		237 (38.9)
No	266 (60.5)	106 (62.7)		372 (61.1)
**Previous smoking cessation attempts**			**0.009**	
No	231 (52.5)	70 (41.1)		301 (49.4)
Yes	209 (47.5)	99 (58.6)		308 (50.6)
**Quitting intention**			**0.020**	
Yes	202 (45.9)	94 (55.6)		296 (48.6)
No	238 (54.1)	75 (44.4)		313 (51.4)

RMB: 1000 Chinese Renminbi about US$160.

**Figure 2:**
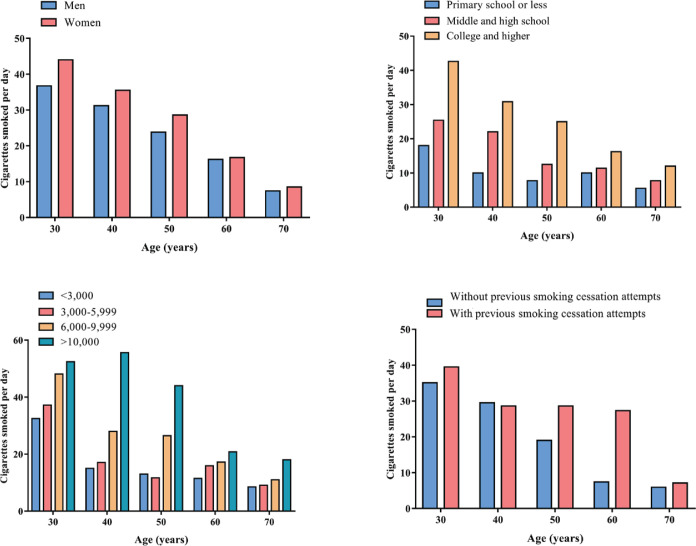
Prevalence of ever use of e-cigarettes in different subgroups

The participants with younger age were more likely to have ever used e-cigarettes, ranging from 37.5% in 18–29 years age group to 6.5% in 60–69 years age group. In addition, across different age groups, the ever use of e-cigarettes was similar between men and women (p<0.05) (Figure 2); the participants with higher levels of education were more likely to have ever used e-cigarettes, ranging from 6.7% among participants who only completed primary school or less to 34.2% among participants who completed college or higher (p<0.05) (Figure 2); the participants with higher levels of monthly income were more likely to have ever used e-cigarettes, ranging from 26.9% among participants whose monthly income was less than 3000 RMB (1000 Chinese Renminbi about US$160) to 42.7% among participants whose monthly income was more than 10000 RMB (p<0.05) (Figure 2); the ever use of e-cigarettes was higher among those who tried to quit smoking than those who never tried to quit smoking (31.9% vs 23.1%, p<0.05) (Figure 2).

After controlling for demographic characteristics, smoking habits and nicotine dependence, as shown in Table 2, the ever use of e-cigarettes was significantly associated with younger age, higher education level, higher monthly income, previous smoking cessation attempts and quitting intention.

**Table 2:** Adjusted ORs of ever use of e-cigarettes in study population

*Variables*	*Model 1 AOR (95% CI)*	*p*	*Model 2 AOR (95% CI)*	*p*
**Gender**				
Male (Ref.)	1		1	
Female	1.104 (0.588–2.073)	0.759	1.039 (0.524–2.061)	0.912
**Age** (years)				
60–69 (Ref.)	1		1	
50–59	2.816 (0.621–12.756)	0.179	1.641 (0.328–8.217)	0.639
40–49	4.594 (1.038–20.324)	0.044	2.922 (0.593–14.413)	0.252
30–39	5.943 (1.317–26.809)	0.020	3.694 (0.734–18.595)	0.156
18–29	8.700 (6.027–10.332)	0.004	12.102 (8.161–16.769)	0.009
**Ethnicity**				
Han (Ref.)	1		1	
Other	1.363 (0.798–2.328)	0.257	1.590 (0.884–2.862)	0.122
**Education level**				
Primary school or less (Ref.)	1		1	
Middle and high school	3.648 (1.079–12.339)	0.037	2.510 (0.669–9.421)	0.202
College and higher	7.266 (2.207–23.916)	0.001	4.343 (1.172–16.101)	0.035
**Monthly income** (RMB)				
<3000 (Ref.)	1		1	
3000–5999	0.765 (0.484–1.210)	0.252	0.961 (0.572–1.615)	0.812
6000–9999	1.064 (0.634–1.788)	0.814	1.385 (0.754–2.541)	0.407
>10000	2.029 (1.186–3.471)	0.010	2.765 (1.469–5.206)	0.003
**Chronic disease**				
Yes (Ref.)	1		1	
No	1.247 (0.764–2.034)	0.377	1.361 (0.766–2.419)	0.293
**Overall satisfaction of life**				
Happy (Ref.)	1		1	
Moderate	0.938 (0.375–2.346)	0.892	1.108 (0.413–2.972)	0.880
Not Happy	1.345 (0.560–3.234)	0.507	1.632 (0.634–4.204)	0.157
**Alcohol use**				
Yes (Ref.)	1		1	
No	1.054 (0.691–1.609)	0.806	1.076 (0.680–1.702)	0.754
**Secondhand smoke exposure**				
Yes (Ref.)	1		1	
No	1.095 (0.760–1.577)	0.628	1.091 (0.735–1.619)	0.666
**Previous smoking cessation attempts**				
No (Ref.)	1		1	
Yes	1.562 (1.091–2.235)	0.015	1.552 (1.055–2.282)	0.026
**Cigarettes smoked per day**				
1–9 (Ref.)	1		1	
10–19	1.201 (0.785–1.838)	0.284	1.294 (0.815–2.054)	0.083
≥20	0.735 (0.470–1.150)	0.162	0.697 (0.431–1.127)	0.099
**Smoking duration** (years)				
1–9 (Ref.)	1		1	
10–19	1.391 (0.845–2.291)	0.194	1.332 (0.769–2.306)	0.206
≥20	1.454 (0.965–2.192)	0.073	1.402 (0.872–2.254)	0.109
**Nicotine dependence**				
Yes (Ref.)	1		1	
No	1.481 (0.820–2.150)	0.157	1.383 (0.900–2.126)	0.184
**Quitting intention**				
No (Ref.)	1		1	
Yes	1.432 (1.084–2.121)	0.000	1.344 (1.158–1.952)	0.003

Model 1 adjusted for gender and age. Model 2 adjusted for gender, age, ethnicity, education level, monthly income, chronic disease, self-reported overall satisfaction of life, and alcohol use, nicotine dependence, previous smoking cessation attempts and secondhand smoke exposure. RMB: 1000 Chinese Renminbi about US$160.

Lastly, to identify the role of socioeconomic status in the pathway between quitting intention and the ever use of e-cigarettes, we performed Sobel tests to estimate how much of the effect is mediated through the channel of socioeconomic status. As shown in Table 3, education level is confirmed as a mediating factor, and approximately 42.86% of the effects on ever use of e-cigarettes were mediated through the channel of higher socioeconomic status.

**Table 3:** Sobel test of mediation for education and income from ever use of e-cigarettes to quitting intention

*Sobel test*	*Total effect*	*Direct effect*	*Indirect effect*	*Proportion of total effect that is mediated (%)*
**Model 1**				
Quitting intention → education → ever use of e-cigarettes	0.058	0.033	0.025	43.10
Quitting intention → income → ever use of e-cigarettes	0.058	0.054	0.004	6.89
**Model 2**				
Quitting intention → education → ever use of e-cigarettes	0.042	0.024	0.018	42.86
Quitting intention → income → ever use of e-cigarettes	0.042	0.034	0.003	6.54

Model 1 only included independent variable (ever use of e-cigarettes), dependent variable (quitting intention), and mediate variable (socioeconomic status). Model 2 additionally included control variables (gender, age, ethnicity, chronic disease, self-reported overall satisfaction of life, alcohol use, nicotine dependence, previous smoking cessation attempts and secondhand smoke exposure).

## DISCUSSION

To the best of our knowledge, this is the first study to examine the possible mediators between cigarette cessation intention and e-cigarette use in a Chinese smoking population. Our findings provided valuable evidence to better understand e-cigarette use patterns and risk factors among the Chinese smoking population.

First, our study found that 3.94% of current smokers were using e-cigarettes, which was very similar to the 3.68% in a large nationally-representative cross-sectional study of 373781 Chinese adults^17^, 3.9% in a citywide representative survey of 10233 Chinese adults^18^, and 3.6 % in a study of 31151 urban Chinese adults^8^, but slightly higher than the 3.0% in the 2018 Global Adults Tobacco Survey China Project^6^. More importantly, the e-cigarette use among current smokers is significantly higher in young people, which is in line with what has been observed in most previous studies^19-21^. This finding is troubling as there is an increasing body of evidence showing that young people who use e-cigarettes will increase their chance of smoking combustible cigarettes later in life by two- to four-fold^22^.

Second, our study showed that some patterns of e-cigarette use in China differed from those in other countries. Particularly, previous nationally representative studies in the USA^23^ and the UK^24^ have indicated that e-cigarette use was highest among those with lower socioeconomic status. Interestingly, our study showed that e-cigarette use in China was more accepted by individuals with higher socioeconomic status, which is consistent with previous studies in Chinese population. Moreover, our study identified high socioeconomic status, particularly higher education level, as a major mediating factor. This heterogeneity in e-cigarette use by socioeconomic status may be explained by different perceptions of e-cigarettes and reasons for using e-cigarettes across countries. Higher socioeconomic status, particularly higher education level, may not only increase the awareness of harmful effects of smoking, but also increase the likelihood of cessation attempts. In fact, several studies have noted that highly educated adults were more likely to quit smoking. In China, the most common reason for using e-cigarettes in China was smoking cessation^19^. Therefore, individuals with higher socioeconomic status might use e-cigarettes to quit cigarette smoking. Furthermore, e-cigarette companies often promote their products as ‘modern and fashionable’ accessories, which make e-cigarettes even more attractive to people with high social class as they are early adopters of new technologies, according to the Diffusion of Innovation theory^25^.

Last, an important debate regarding e-cigarettes is whether they can be used as an aid for smoking cessation. Although this study did not directly assess the efficacy of e-cigarettes on smoking cessation, we found that e-cigarettes are more attractive to current smokers who have tried to quit smoking, which was consistent with most previous research. However, regulatory authorities have yet to confirm such claims. World Health Organization stated in 2019 that there is ‘insufficient independent evidence to support the use of e-cigarettes as a population-level tobacco cessation intervention to help people quit conventional tobacco use’^26^. More importantly, no clinical research regarding e-cigarettes for smoking cessation has been reported in China. We strongly recommend that well-designed randomized controlled clinical trials be conducted to assess the clinical efficacy of e-cigarettes as a smoking cessation aid compared with approved smoking cessation therapies.

### Strengths and limitations

The major strengths of this study are the stringent quality control processes to ensure the validity and reliability of our study findings. However, our study has several limitations. First, this was a cross-sectional study, which is unable to establish causal relationships. Second, we did not include individuals aged ≤18 years, thus we cannot provide information on teenagers who might also have used e-cigarettes. Third, e-cigarettes and smoking use in this study were self-reported, which may be affected by recall bias. Fourth, our study was conducted in major urban cities and may not be generalizable to other cities or rural areas in China. Last, most of the subjects were men.

## CONCLUSIONS

To the best of our knowledge, this was the first study to examine the possible mediators between cigarette cessation intention and e-cigarette use in a Chinese smoking population. The findings revealed that high socioeconomic status, particularly higher education level, was a major mediating factor.

## Data Availability

The data supporting this research are available from the authors on reasonable request.
